# Component-Resolved Diagnosis Based on a Recombinant Variant of Mus m 1 Lipocalin Allergen

**DOI:** 10.3390/ijms24021193

**Published:** 2023-01-07

**Authors:** Elena Ferrari, Daniela Breda, Alberto Spisni, Samuele E. Burastero

**Affiliations:** 1 Department of Medicine and Surgery, University of Parma, 43126 Parma, Italy; 2 Division of Immunology, Transplantation and Infectious Diseases, San Raffaele Scientific Institute, 20132 Milan, Italy

**Keywords:** occupational allergy, recombinant allergen, allergen component, allergen extract, Major Urinary Protein, Mus m 1, IgE-ELISA, conformational epitopes

## Abstract

Exposure to the Mus m 1 aeroallergen is a significant risk factor for laboratory animal allergy. This allergen, primarily expressed in mouse urine where it is characterized by a marked and dynamic polymorphism, is also present in epithelium and dander. Considering the relevance of sequence/structure assessment in protein antigenic reactivity, we compared the sequence of the variant Mus m 1.0102 to other members of the Mus m 1 allergen, and used Discotope 2.0 to predict conformational epitopes based on its 3D-structure. Conventional diagnosis of mouse allergy is based on serum IgE testing, using an epithelial extract as the antigen source. Given the heterogeneous and variable composition of extracts, we developed an indirect ELISA assay based on the recombinant component Mus m 1.0102. The assay performed with adequate precision and reasonable diagnostic accuracy (AUC = 0.87) compared to a routine clinical diagnostic test that exploits the native allergen. Recombinant Mus m 1.0102 turned out to be a valuable tool to study the fine epitope mapping of specific IgE reactivity to the major allergen responsible for mouse allergy. We believe that advancing in its functional characterization will lead to the standardization of murine lipocalins and to the development of allergen-specific immunotherapy.

## 1. Introduction

Laboratory animal allergy (LAA) is a relevant occupational health hazard for workers attending veterinary or research animal facilities. It was estimated that as many as 44% of personnel operating in contact with laboratory animals report symptoms related to occupational asthma or allergic rhinoconjunctivitis [1,2].

Since animal allergens can associate with dust particles and remain airborne for a period of time, aeroallergen exposure is considered the most significant risk factor for LAA [3]; among these inhalant allergen sources are mouse allergens, including Mus m 1 (WHO/IUIS Allergen Nomenclature Database).

Mus m 1 is present in mouse epithelium, urine, serum, saliva, hair, and dander [4]. Urine is the principal source of the major mouse allergen identified as the Mouse Major Urinary Proteins (MUPs) family. These proteins are lipocalins, whose functional signature is pheromonal communication. They are capable of binding to semiochemicals with their hydrophobic pocket and delivering them to conspecifics, signalling a wide range of information [5,6]. MUPs are encoded by a cluster of 22 MUP genes located on chromosome 4. They can be split into two categories, termed peripheral and central genes (coding peripheral and central MUPs, respectively) according to their position along the chromosome. Individual MUP urinary profiles derive from a combination of variation in sequence and differential transcription of central MUP genes [5,7,8,9].

The three-dimensional structure of an allergen defines the epitopes that may interact with antibodies. In the sensitization phase of allergic diseases, B-cells are stimulated to produce IgE molecules against specific conformational epitopes exposed on the allergen surface. During a subsequent exposure to the same allergen, the recognition of those epitopes by IgE antibodies bound to specific sensitized effector cells results in clinically relevant outcomes of variable severity, even including anaphylactic reactions [10].

In vitro diagnosis of mouse allergy is based on the detection of allergen-specific serum IgE (sIgE) using an extract of epithelial source as allergen [11,12]. While an extract of natural origin can be a complex mixture of allergenic and non-allergenic substances, several single allergen components, mainly produced as recombinant proteins, are now available for component-resolved diagnosis [13,14,15,16,17,18].

Recombinant Mus m 1.0102 protein (rMus m 1.0102) is a member of the MUPs family and has been included with the Mus m 1 allergen as an allergen component [6,19,20]. In addition to disclosing its structural features, our research group has demonstrated its ability to react with IgE from mouse-allergic patients and cause a dose-dependent degranulation response in rat basophilic leukemia cell line [6,20,21].

While various studies have investigated the role of allergen molecules in the diagnosis of occupational allergens [22,23,24], sIgE reactivity to specific Mus m 1 components has only been limitedly explored [25]. In the present work, (i) we used bioinformatic tools to compare the rMus m 1.0102 sequence to other known members of the MUPs cluster and to predict rMus m 1.0102 conformational epitopes; and (ii) we set up a diagnostic tool based on recombinant Mus m 1.0102 which may allow us to distinguish the fine specificity of IgE binding to a single component from cross-reactive proteins.

Our approach, by determining the features of the allergen-antibody interactions, paves the way to the introduction of this allergen in molecular allergology.

## 2. Results

### 2.1. Bioinformatic Approach to Mus m 1.0102 Protein

Recognizing the relevance of sequence/structure assessments in protein antigenic reactivity, (i) we searched for sequence similarities among mouse MUPs and Mus m 1.0102; and (ii) we performed a prediction of rMus m 1.0102 conformational epitopes based on its three-dimensional structure.

#### 2.1.1. Sequence Comparison with MUP Isoforms

**Figure 1:**
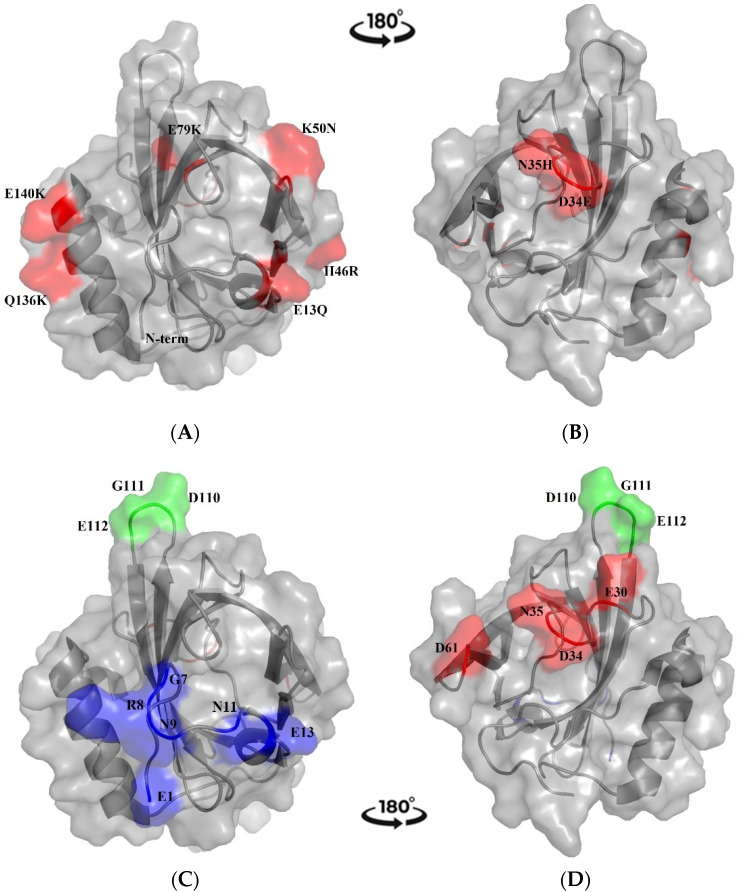
Three-dimensional structure views of the rMus m 1.0102 allergen (PDB code 1df3, model 1): (**A**,**C**) bottom view of the central cavity, (**B**,**D**) top view of the cavity, generated by a 180° turn. (**A**,**B**) Surface and cartoon representations of the rMus m 1.0102 structure. The amino acid substitutions observed in central MUP isoforms correspond to surface-exposed residues of rMus m 1.0102 (red). (**C**,**D**) Coloured residues of surface and cartoon representations of rMus m 1.0102 solution structure correspond to the three B-cell epitopes predicted by Discotope 2.0 (epitope I in blue, epitope II in red, and epitope III in green).

We performed a BLAST sequence similarity search on the UniProt resource platform [26] and obtained 28 hits, representing the entire Major Urinary Protein family. The sequence alignment in Figure S1 includes Mus m 1.0102 and the MUPs with the best sequence similarity ratings; overall, this selection comprises only central MUPs sequences. According to the alignment, in general, the selected central MUPs differ from the Mus m 1.0102 sequence by two/three residues, with only one MUP differing by five residues. All these variants, as compared to Mus m 1.0102, involve a substitution with (i) a residue with the same charge, (ii) a residue with opposite charge, or (iii) a charged residue instead of a polar residue and vice versa. In Figure 1, the substitutions are displayed on the solution structure of rMus m 1.0102. Clearly, they are located on the surface of the protein. The only exception is for the substitution F56V (not shown in Figure 1), affecting a residue whose side chain is in the central hydrophobic cavity.

However, when aligning the Mus m 1.0102 sequence with the peripheral MUPs pinned down by the BLAST search (Figure S2), we observed an increased number of substitutions, including those between amino acids with little or no similarity, according to scoring matrix PAM 250 for sequence comparison [27]. These are expected to affect residues located on the surface as well as in core regions of the protein.

#### 2.1.2. Prediction of B-Cell Conformational Epitopes

The Discotope 2.0 server was used to predict discontinuous B-cell epitopes from the Mus m 1.0102 three-dimensional structure (PDB code 1df3, model 1) [28]. The output of the prediction is presented in Figure S3 and Table S1. The predicted epitopes are displayed on the rMus m 1.0102 solution structure in Figure 1C,D. The prediction includes three surface areas corresponding to epitope I (blue, Figure 1C), epitope II (red, Figure 1D), and epitope III (green, Figure 1C,D). Residues of epitope I belong to the N-terminal region, those of epitope II to the loop between the β-strands A and B, and those of epitope III to the loop between the β-strands G and H [29].

Epitope prediction, based on the Mus m 1.0102 three-dimensional structure (PDB code 1df3, model 1), was also generated by peer methods. The SEPPA 3.0 prediction includes 26 epitope residues (Table S2 and Figure S4), covering a larger surface area as compared to the Discotope 2.0 prediction (Figure 1). Notably, those epitope regions include residues R8, D61, D110, G111, and E112 that were also predicted by Discotope 2.0 as parts of the conformational epitopes reported in Figure 1C,D. The C-terminal epitope region (Figure S4B) was predicted only by SEPPA 3.0. Similarly, the SEMA prediction (not shown) includes several residues predicted also by Discotope 2.0 and SEPPA 3.0.

**Figure 2:**
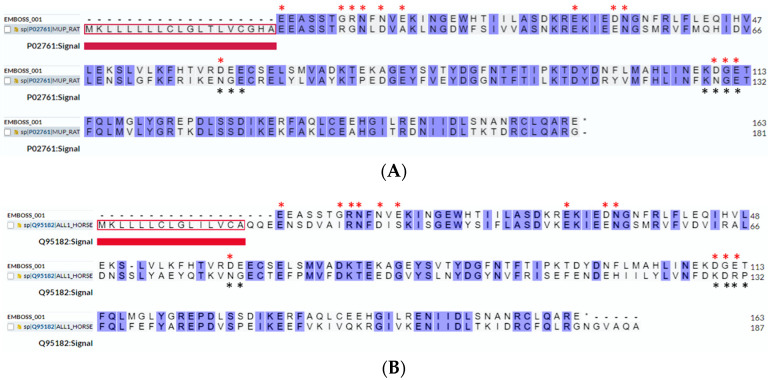
Sequence alignment of rMus m 1.0102 with cross-reactive allergens (blue coloured by identity on the UniProt platform). Recombinant Mus m 1.0102 is aligned with (**A**) Rat n 1, and (**B**) Equ c 1 allergens. Asterisks indicate residues involved in B-cell epitopes of rMus m 1.0102 (red) and Rat n 1 or Equ c 1 allergens (black), as predicted by the Discotope 2.0 server. Proteins are represented by their accession numbers in the UniProtKB database, while the query protein Mus m 1.0102 is indicated as EMBOSS_001 (dashed line at the beginning of the sequence indicates omission of the secretion signal). Residues in the red box are secretion signals (thick red lines).

B-cell epitope prediction by Discotope 2.0 was performed also on the 3D-structure of Rat n 1 and Equ c 1 lipocalin allergens (PDB codes 2A2U and 1EW3, respectively), mammalian lipocalins characterized by IgE cross-reactivity [30,31]. Residues of predicted epitopes are highlighted in the sequence alignments with Mus m 1.0102 in Figure 2.

We conclude that: (i) few residues differentiate Mus m 1.0102 from central MUPs, mainly corresponding to Mus m 1.0102 solvent-exposed residues; (ii) major differences in surface composition and core interactions are observed when comparing Mus m 1.0102 with peripheral MUPs; (iii) rMus m 1.0102-predicted epitopes involve mainly polar and charged loop residues, exposed on the protein surface and presumably affected by fewer structural constraints compared to secondary structure regions; and (iv) rMus m 1.0102 structure shares predicted conformational epitopes with cross-reactive Rat n 1 and Equ c 1 allergens.

### 2.2. Preparation of Recombinant Mus m 1.0102 and Urinary Protein Samples

Recombinant Mus m 1.0102 protein, expressed in *Pichia pastoris*, is secreted in the yeast supernatant [20]. Taking advantage of this preliminary protein separation, an anion-exchange column was sufficient to isolate rMus m 1.0102 from the yeast supernatant. The elution fractions turned out to contain virtually pure recombinant protein, as indicated by the band of approximately 20 kDa in SDS-PAGE analysis (Figure S5A). Its identity was validated using a rabbit anti-MUP polyclonal antibody (Figure S5B). Characterization of the secondary structure composition of this protein sample was performed with circular dichroism (CD) spectroscopy; the far-UV CD spectrum in Figure S5C confirms the secondary structure composition previously attributed to that protein isoform [20].

A sample of native urinary protein (nMus m 1), prepared as previously described [32], was a kind gift of Prof. Cavaggioni’s Group. SDS-PAGE electrophoresis and Western blotting showed a major protein band centered around 20 kDa (Figure S5A,B) that we associated with nMus m 1 [33,34]. However, because of the low-resolution preparative protocol (see Section 4.2), the presence of different protein isoforms in the final preparation cannot be excluded. The far-UV CD spectrum of nMus m 1 (Figure S5C) demonstrated good secondary structure similarity to rMus m 1.0102.

Based on these results, we assume that both protein samples can be used in an ELISA immunoassay.

### 2.3. Indirect ELISA Immunoassay

Indirect ELISA was used to evaluate the reactivity of sIgE antibodies from Mus m 1 sensitive subjects against the rMus m 1.0102 protein. The results were compared with assays performed with two samples of nMus m 1; a laboratory (see Section 2.2) and a commercial (see Section 4.2) preparation of the murine urinary allergen. Preliminarily, we carried out a quantitative assay measuring sIgE binding to commercially available mouse allergens, including Mus m 1 allergen of epithelial origin. This last step, demonstrating IgE reactivity in the routine setting of the clinical lab, was used as a gold standard for mouse allergy diagnosis.

#### 2.3.1. Allergy Diagnosis by ImmunoCAP ISAC Test

The ImmunoCAP ISAC test is a multiplex platform that simultaneously measures individual IgE reactivity to 112 allergen components that can trigger allergic reactions [35]. The test was performed to select the sera of allergic subjects with IgE binding to native Mus m 1 of epithelial origin. Control sera from healthy subjects gave negative results to the ImmunoCAP ISAC test, validating our experimental setup.

Native allergen components included in the ISAC panel, such as Mus m 1, are glycoproteins. Reactivity to the carbohydrate determinants of native allergen may variably account for the observed IgE binding, a fact which is not associated with clinically relevant allergic reaction in vivo. To take into account that possible interference, ImmunoCAP ISAC includes one reagent that quantifies reactivity to carbohydrate determinants (CCD). In particular, the MUXF3 CCD reagent contains the carbohydrate epitope purified from digested bromelain (Ana c 1), a glycoprotein from pineapple (*Ananas comosus*). The MUXF3 carbohydrate chain is found in many plant proteins, and IgE to MUXF3 is highly cross-reactive in different allergen sources [36].

As a further surrogate marker of IgE reactivity to carbohydrates, we included the native component Cup a 1 from *Cupressus arizonica* since native allergens from Cupressaceae in general [37] and native Cup a 1 in particular [38] are known to be heavily glycosylated.

Finally, the analysis of IgE reactivity to murine lipocalins was integrated with the measurement of sIgE antibodies to lipocalins from horse (Equ c 1), dog (Can f 1) and cat (Fel d 4), based on the structural similarity of these allergens and cross-reactivity of lipocalin-specific IgE (Table 1).

#### 2.3.2. Indirect ELISA Immunoassay with Recombinant and Urinary Mus m 1 Samples

**Figure 3:**
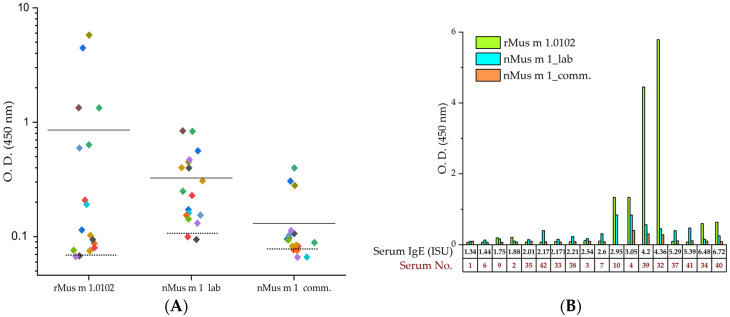
Indirect ELISA for the detection of IgE antibodies binding to Mus m 1 allergen from different sources. (**A**) Binding signals of recombinant Mus m 1.0102 and nMus m 1 samples (laboratory and commercial preparations) to serum IgE molecules are presented as O.D. at 450 nm. Discrete values are displayed in different colours according to the allergic sera, with the bold horizontal line representing the mean O.D. value. The dashed lines represent the mean signal obtained with control sera in each ELISA test. The distributions of each ELISA dataset obtained with IgE-positive and control sera are significantly different (Mann–Whitney test). *y* axis is in log_10_ scale. (**B**) Results of the indirect ELISA are reported as a function of increasing Mus m 1-specific IgE concentration (measured by ImmunoCAP ISAC test). Volume of serum No. 10 was insufficient for testing with Mus m 1 commercial preparation.

In the indirect ELISA, rMus m 1.0102 as well as nMus m 1 laboratory and commercial preparations were incubated separately with IgE-positive and control sera. The serum IgE-reactivity profile of positive sera, resulting from three ELISA assays, is presented in Figure 3. Positive sera yielded mean optical density (O.D.) values of 0.854 ± 1.62 for rMus m 1.0102, and 0.325 ± 0.23 and 0.130 ± 0.10 for the laboratory and commercial preparations of nMus m 1, respectively (Figure 3A).

The panel of 18 IgE-positive sera revealed measurable IgE reactivity to rMus m 1.0102 in 88.9% of sera (16 positive sera out of 18), equalling the nMus m 1 laboratory sample (Table 2). The nMus m 1 commercial sample had a performance below 80%, possibly because of a partial degradation process (Figure S5A).

A correlation analysis was performed to compare the ELISA results obtained with rMus m 1.0102 with the data obtained with the other protein reagents (Table S3). Spearman correlation was significant at the 0.05 level only for the comparison with the nMus m 1 laboratory preparation (r = 0.51496). A significant correlation was found also when comparing the ELISA data obtained with rMus m 1.0102 with the IgE levels expressed in ISU (r = 0.55005).

#### 2.3.3. ROC Analysis Based on Indirect ELISA Data

Receiver operator characteristics (ROC) analysis was performed on the ELISA results obtained with rMus m 1.0102 and urinary nMus m 1 samples by applying the ImmunoCAP ISAC test response for diagnosis (positive or negative). Accuracy indices (area under the curve [AUC], *p*-value) for the ROC curves were obtained from the analysis (Table 3). Since the *p*-values are much smaller than 0.05, we can conclude that the different approaches may still be effective. The threshold O.D. values were determined by the corresponding ROC curves (Table 3, Figure S6); by balancing optimal Sensitivity and Specificity, they correspond to the O.D._450nm_ value above which serum can be considered positive, based on the indirect ELISA test.

### 2.4. Precision of the ELISA Immunoassay Based on Recombinant Mus m 1.0102 Allergen

To evaluate the precision from repeated ELISA measures based on rMus m 1.0102, we calculated the inter-assay and the intra-assay coefficients of variability (CV). Serum samples with high, medium, and low IgE concentration (according to ImmunoCAP ISAC test) were used in five replicates to calculate the CV percentage. Intra-assay CV% ranged from 1.8% to 6.5% and the inter-assay CV% ranged from 4.1% to 9.1% (Table 4), reflecting data reliability.

## 3. Discussion

Mouse allergen extracts, i.e., aqueous extraction of compounds obtained from natural sources, may be used for the diagnosis and, potentially, for allergen-specific immunotherapy of mouse allergy. Biologic potency and standardization of allergen content are well-known critical issues [39]. In different mouse epithelial allergen extracts, total and isoform specific MUP-quantification turned out to be variable and low, while urine samples revealed a different composition in terms of isoforms and concentrations when compared with commercial epithelial extracts [40,41]. Because of all these discrepancies, the use of epithelial extracts in the diagnosis of mouse allergy has been a matter of debate. Moreover, since commercial mouse epithelial extracts derive mainly from hair dander of laboratory strains, they may in principle be more appropriate for the diagnosis and treatment of animal facility workers than for individuals reactive to domestic mouse infestations.

The allergic subjects participating to this study were suffering from occupational mouse allergy and clinical diagnosis correlated with positive a ImmunoCAP ISAC test, detecting Mus m 1-specific IgE molecules to a native allergen component of epithelial origin.

We show here that the use of the rMus m 1.0102 allergen may represent an advancement for mouse allergy diagnosis. The use of several recombinant MUPs in serology vs. native mouse allergen has been previously investigated [25]. Recombinant isoforms (from MUP1, MUP2, MUP4, MUP7, MUP8 and MUP9 genes) exhibited similar antigenicity as a native Mus m 1 allergen, and a high degree of cross-reactivity with Mus m 1 for both IgE and IgG molecules. Those findings are in line with our results.

Our bioinformatic analysis established that the Mus m 1.0102 sequence best ranks among the central MUPs group, characterized by >97% sequence homology. The few substitutions emerging from its alignment with central MUPs (Figure S1) turned out to involve residues located on the surface of the rMus m 1.0102 structure, in accordance with previous works [5,8,9]. Those surface substitutions (i) may modify the net charge of the molecule, and (ii) might influence the mechanisms that govern not only the interaction with MUPs vomeronasal receptors [42] but also with antibodies directed against the protein itself. We emphasize that these findings attribute a unique amino acid composition, and possibly a peculiar arrangement of antigenic determinants, to the rMus m 1.0102 surface. This feature is expected to impact on the ability to bind sIgE molecules of mouse allergic subjects. In contrast, the comparison with peripheral MUPs showed a greater degree of sequence heterogeneity (Figure S2), presumably affecting both inner and outer structural regions, thus potentially influencing different functional properties.

Epitopes may correspond to protein linear fragments or exhibit a specific conformation. Moreover, it is expected that B-cell conformational epitopes involve discontinuous amino acids in the protein sequence that become contiguous in the folded tertiary structure. Prediction of these determinants may contribute to the identification of putative IgE epitopes. Discontinuous B-cell epitopes were predicted by Discotope 2.0 [43], a tool of the Immune Epitope Database (IEDB) Analysis Resource. With a default threshold score of −3.7, the Discotope output for the rMus m 1.0102 3D-structure revealed regions of positive prediction (Figure S3, Table S1). They identify two surface patches located at two opposite sides of the structure (bottom and entrance of the central cavity, epitopes I and II), and a third one in a protruding loop connecting two β-strands (epitope III) (Figure 1C,D). The latter was predicted also when the threshold score was considerably increased (up to +0.5 value, allowing for 90% specificity and 23% sensitivity). Compared to Discotope 2.0, SEPPA 3.0 results involve an increased number of residues (Table S2). However, some of the predicted epitope regions include key residues predicted also by Discotope 2.0 (Figure S4) and SEMA. We conclude that we might have identified robust antigenic regions of the protein surface that need to be experimentally verified in future studies.

Overall, epitope predictions imply that rMus m 1.0102 has antigenic properties [44], a prerequisite for allergenicity and specific IgE-binding ability. Moreover, epitope III (predicted by Discotope 2.0) is shared by Rat n 1 and Equ c 1, which may explain the occurrence of cross-reacting sIgE in patients primarily allergic to mice [45,46]. Interestingly, among the residues involved in the predicted epitopes, E13, D34, and N35 correspond to residues which undergo substitution both in central (Figure 1A,B) and peripheral MUPs (Figures S1 and S2); this fact reinforces the idea that rMus m 1.0102 may represent a component endowed with specific antigenicity. This property could impact on the capability of rMus m 1.0102 to discriminate between genuine IgE-based sensitization to Mus m 1 and cross-reactivity to Rat n 1 and Equ c 1. Due to the limitations of current reagents, IgE-binding inhibition experiments with soluble allergens, aimed to discriminate and quantify genuine sensitization from cross-reactivity, could not be carried out. However, by combining bioinformatic analysis with comparison of IgE-binding scores in different assays, our results indicate that rMus m 1.0102 can be a useful tool to investigate fine specificity differences of IgE binding to lipocalins from different species.

The cohort of patients we studied was primarily allergic to mice. In 6 out of 18 patients, sIgE to more than one lipocalin of other species (cat, dog, or horse) was measurable, albeit at lower titres compared to IgE to Mus m 1, likely due to cross-reactivity. Recombinant Mus m 1.0102 could not discriminate mouse lipocalin binding more efficiently than the native Mus m 1 included in the ISAC panel (not shown).

In contrast, in the indirect ELISA assay, rMus m 1.0102 proved to be a valuable alternative to mouse epithelial extracts for specific IgE-antibody testing. In fact, among the allergic sera, the prevalence of IgE reactivity to rMus m 1.0102 reached 88.9%, equivalent to the laboratory preparation of nMus m 1, presumably enriched in most abundant MUP isoforms. In addition, the calculated coefficients of variability revealed an acceptable degree of precision of the recombinant allergen, thus minimizing inconsistencies among assay replicates.

Considering all the ELISA assays performed, rMus m 1.0102 non-reactive sera matched with those characterized by lower Mus m 1-specific IgE levels as measured by ISAC (Table 1 and Table 2). In particular, the diagnostic sensitivity of rMus m 1.0102 considerably increased with specific IgE levels higher than 2.95 ISU (Figure 3B). However, relatively low reactivity to rMus m 1.0102 was also measured in sera 37 and 41, which scored high in IgE to native Mus m 1 in ISAC test. Notably, in the corresponding subjects, a positive reactivity to both MUXF3 and Cupressaceae was detected, suggesting reactivity to the carbohydrate moiety of allergens [47]. We postulate that the contamination by CCD of the native Mus m 1 component in ImmunoCAP ISAC test might explain this discrepancy.

As for the allergic sera 34 and 40, which scored negative for IgE to CCDs, their reactivity to rMus m 1.0102 as well as to nMus m 1 samples was moderate, despite their high level of Mus m 1-specific IgE in the ISAC analysis, suggesting that the IgE repertoire in those sera was reactive to epitopes differentially expressed in the compared reagents.

Six out of 18 allergic sera—characterized by the highest measured Mus m 1-specific sIgE levels—reacted to rMus m 1.0102, producing significantly higher responses compared to the nMus m 1 samples (Figure 3B, serum No. 4, 10, 32, 34, 39 and 40). These results suggest that, as demonstrated for routine diagnostics [48,49], the use of a non-glycosylated recombinant Mus m 1 allergen might improve diagnostic sensitivity, without introducing reactivity to the cross-reactive CCD moiety.

Furthermore, it is feasible that the rMus m 1.0102 allergen may represent the clonally prevalent fine specificity of the polyclonal repertoire of antibodies to mouse allergens. Indeed, nMus m 1 purified from laboratory mice urine is expected to be enriched in most abundant MUP isoforms, but it may include other allergenic and/or non-allergenic protein contaminants. That possibility would decrease the epitope density on the assay solid phase, yielding lower binding signals with sera in which rMus m 1.0102-specific reactivity was clonally prevalent.

## 4. Materials and Methods

### 4.1. Bioinformatic Analysis of Mus m 1.0102 Sequence and Protein Structure

We used Basic Local Alignment Search Tool (BLAST) for the Mus m 1.0102 sequence similarity search in the UniProt protein database, setting the advanced parameters to E-Threshold = 10 and Matrix = BLOSUM62. We narrowed the search to “mouse” and a sequence length of 1–200 residues. The Align tool, running the Clustal Omega algorithm to find areas of identity/similarity [50], was used for multiple sequence alignment.

The Discotope 2.0 server was used to predict discontinuous B-cell epitopes from protein three-dimensional structures. The method utilizes the calculation of surface accessibility (estimated in terms of contact numbers) and a specific epitope-propensity amino-acid score. Final scores are determined by combining the propensity scores of residues in spatial proximity and the contact numbers [43,51]. The threshold score for epitope prediction was set at −3.7 (default value), which corresponds to a specificity of 75% and a sensitivity of 47%; residues with a score above the threshold were predicted to be parts of epitopes.

SEPPA 3.0 and SEMA servers also were used for computational identification of immunogenic regions in the rMus m 1.0102 structure.

### 4.2. Preparation of Urinary and Recombinant Mus m 1 Samples

Major Urinary Proteins were separated from male mouse urine in the laboratory of Prof. Cavaggioni’s Group at University of Parma. Urine collection was authorized by the Italian Ministry of Health (number 154/2003B) and was performed according to the Italian and European laws on animal experiments and welfare. Purification of urinary proteins was based on their protein size and charge at pH 7.2, assuming a molecular mass in the range 17–20 kDa and an isoelectric point of about 4.8. Briefly, urinary proteins were separated by ammonium sulphate precipitation; next, MUPs fractionation was performed by low-pressure chromatography, using sequentially a size-exclusion column (Sephadex G-50, Merck, Darmstadt, Germany) and an anion-exchange column (Whatman anion exchange cellulose DE52, Merck, Darmstadt, Germany) [32]. Protein elution was monitored by UV protein absorbance at 280 nm, and protein-containing fractions were analyzed with sodium dodecyl sulphate-polyacrylamide gel electrophoresis (Tris-Glycine SDS-PAGE, 12%) in reducing conditions. MUP-containing fractions were combined, dialyzed against 20 mM phosphate buffer pH 7.2, and lyophilized. When powder was made up into a solution, protein concentration was determined spectrophotometrically, assuming an extinction coefficient ε_278_ = 11,345 M^−1^ cm^−1^. This urinary nMus m 1 sample is referred to as “laboratory preparation” throughout the manuscript.

Molecular cloning of the cDNA encoding Mus m 1.0102 variant into *Pichia pastoris* yeast, expression of the recombinant protein, its purification and characterization are described in references [6,20,21,52]. Briefly, after an extensive dialysis against Tris-HCl 10 mM pH 7.2, 100 mL of the yeast culture supernatant, containing exclusively the secreted recombinant allergen, were filtered through a membrane with 0.45 μm pore size (Millipore membrane filter, Merck, Darmstadt, Germany), concentrated by ultrafiltration (Amicon Ultra-15 centrifugal filter, 10 kDa MWCO, Merck, Darmstadt, Germany ) and separated by anion-exchange chromatography. This last step was performed with an FPLC system (ÄKTA protein purification system, Merck, Darmstadt, Germany) fitted with a Source 15Q 4.6/100 PE column (Thermo Fisher Scientific, Waltham, MA, USA). The column was equilibrated with 10 mM Tris-HCl buffer pH 7.2, before loading the concentrated sample solution (4 mL). Bound protein was eluted from the column using a linear salt gradient (0–1 M NaCl in 10 mM Tris-HCl buffer). Fractions of the unique protein elution peak were collected and dialyzed against 10 mM Tris-HCl buffer pH 7.2. Protein concentration was determined spectrophotometrically, assuming an extinction coefficient ε_278_ = 11,345 M^−1^ cm^−1^.

Urinary and recombinant protein samples were analyzed by SDS-PAGE 12% in reducing conditions. After SDS-PAGE electrophoresis (15 % acrylamide), similar amounts of rMus m 1.0102 and urinary nMus m 1 allergen were electro-blotted onto PVDF membrane and incubated over-night at 4 °C with rabbit anti-MUP polyclonal antibody (MUP FL180 antibody, 1:200 dilution, Santa Cruz Biotechnology, Dallas, TX, USA). Detection was performed with HRP-conjugated anti-rabbit IgG antibodies by chemiluminescence autoradiography.

CD spectra of the recombinant and urinary protein samples were acquired with a JASCO J-715 spectropolarimeter, equipped with a Peltier-controlled cuvette holder. Spectra were collected at a scan speed of 20 nm/min and with a response time of 1 s. Each spectrum was the average of three scans. Far-UV CD spectra were collected at protein concentrations of 4 μM and with a 1 mm path length cell. Data acquired in the range 195–250 nm were graphed as mean molar residue ellipticity [θ], deg cm^2^ dmol^−1^.

Urinary nMus m 1 allergen was also purchased from Indoor Biotechnologies (product code NA-MM1-1, Indoor Biotechnologies, Cardiff, United Kingdom). As reported by the company, it was purified from concentrated male mouse urine by gel filtration and ion-exchange HPLC. Protein purity was estimation by SDS-PAGE to be 95%, as declared by the manufacturer. This urinary nMus m 1 sample is referred to as “commercial preparation” throughout the manuscript.

### 4.3. Serum Samples, Allergy Diagnosis and IgE Quantification

Eighteen serum samples from Mus m 1-sensitized individuals (allergic sera) and ten healthy nonatopic individuals (control sera) were provided by IRCCS Ospedale San Raffaele (Milano, Italy). Informed consent for the use of sera was obtained from all the subjects involved in this study (see ethics clearance below).

Allergic sera (N = 18) were obtained from subjects that had access to a standard pathogen-free animal facility. Following exposition to mouse allergens, they had developed respiratory symptoms which allowed them to receive a clinical diagnosis of allergic rhinitis with or without allergic asthma, according to EAACI guidelines [53]. Sensitization to mouse lipocalin was assessed by the detection of specific IgE to native Mus m 1 (ImmunoCAP ISAC test). None of the patients had previously been diagnosed with allergic rhinitis with or without allergic asthma, nor had any ever reported respiratory symptoms in the presence of pets (in particular, cats, dogs or horses). Therefore, these patients were considered primarily allergic to mice following professional exposure.

Serum samples were separated by centrifugation at 500× *g* for 15 min at room temperature and used for IgE detection. The IgE repertoire of each serum was analyzed using the multiplex ImmunoCAP ISAC test (Thermo Fisher Scientific, Waltham, MA, USA). IgE quantification, as a measure of IgE allergen-specific reactivity, is expressed in arbitrary ISAC Standardized Units (ISU). According to the method, ISU values are classified into four categories: values < 0.3 ISU are defined as negative; values between 0.3 and 1 ISU as low-level positive; values between 1 and 15 ISU as moderately high; values > 15.0 ISU as very high positive. No IgE reactivity (ISU < 0.3) was detected in all the control sera.

### 4.4. Indirect IgE-Enzyme-Linked Immunosorbent Assay (IgE-ELISA)

Serum IgE binding to recombinant and native proteins was detected by indirect IgE-ELISA. According to this technique, the protein antigen is immobilized to the surface of a multi-well plate, sIgE primary antibodies bind to the antigen, and a labelled secondary antibody against human IgE molecules binds to the primary antibodies, allowing for detection.

For this assay, 96-well plates (Pierce™ Protein L Coated Plates, Thermo Fisher Scientific, Waltham, MA, USA) were coated overnight at 4 °C with the native or recombinant antigen (100 μL per well, at the final concentration of 10 μg/mL in 50 mM carbonate/bicarbonate buffer, pH 9.6). After 3 washes in phosphate-buffered saline solution containing 0.05% Tween 20 (PBST), the plates were blocked with 200 μL of blocking buffer (Thermo Fisher Scientific, Waltham, MA, USA), for 2 h at room temperature. After a washing step with PBST, diluted sera (1:20 with PBST containing 0.5% BSA) were loaded on the plates (100 μL) and incubated overnight at 4 °C. Then, plates were thoroughly washed with PBST (5 times). A polyclonal anti-human IgE secondary antibody conjugated to horseradish peroxidase (Thermo Fisher Scientific, Waltham, MA, USA) was diluted 1:4000 in PBST containing 0.5% BSA, applied to each well (100 μL) and incubated for 2.5 h at room temperature in the dark. Following washing with PBST (5 times), bound antibodies were detected by incubation with 100 μL of TMB Substrate Solution (ready-to-use substrate system containing 3,3′,5,5′—tetramethylbenzidine, Thermo Fisher Scientific, Waltham, MA, USA) for 30 min at room temperature and successive acidification with 100 μL of 0.16 M H_2_SO_4_. The reaction was quantified by measuring the absorbance at 450 nm.

All samples were run in duplicate, and the mean optical density (O.D.) value was used for analysis. The cut-off for positive ELISA reactivity was determined using the mean O.D. values obtained with 10 sera of subjects without history of any allergy.

To monitor intra-plate or plate-to-plate variation, intra- and inter-assay Coefficients of Variability (CV) were calculated. Samples were run in quintuplicate on two different plates. CV is calculated by dividing the standard deviation of a set of measurements by the mean of the set and expressed as a percentage. 

### 4.5. Statistical Analysis

Statistical data analysis and graphing were performed using OriginPro, Version 2021 (OriginLab Corporation, Northampton, MA, USA). The Mann–Whitney test was used for evaluating differences between the ELISA data sets obtained with Mus m 1 allergic and control sera. For correlation analysis, the Spearman correlation coefficient, specific for non-normally distributed data, was calculated. Values of *p* < 0.05 or < 0.01 were considered to indicate a statistically significant difference.

The diagnostic performance of each ELISA assay was determined from receiver-operating characteristic (ROC) curves, computed in OriginPro, Version 2021. The threshold value of each ELISA assay was obtained by balancing optimal sensitivity and specificity.

## 5. Conclusions

Based on our data, mouse sensitization was confirmed in a percentage of sera from mouse allergic patients ranging between 76.4% and 88.9%. ROC analysis attributed a good to very good accuracy level to the ELISA assays, with AUC values in the 0.77–0.89 interval. A remarkable diagnostic performance was demonstrated by rMus m 1.0102 (AUC = 0.87) which, together with its extensive structural characterization, makes it an important reagent for epitope mapping and mouse allergy diagnostics. It is worth mentioning that (i) further characterization and standardization of the recombinant allergen [54] might contribute to developing a preventive strategy based on recombinant allergen-specific immunotherapy, and (ii) the predicted conformational epitopes represent a starting point for introducing mutations into rMus m 1.0102 to create candidate hypoallergenic molecules for an allergen-specific immunotherapy that prevents the risk of immediate adverse reactions [55].

## Figures and Tables

**Table 1:** Subject characterization by ImmunoCAP ISAC test.

Serum No.	Specific IgE to Lipocalins and to CCD (ISU Units)					
	Mus m 1	MUXF3	nCup a 1	Equ c 1	Fel d 4	Can f 1
1	1.34	0.00	0.43	0.00	0.00	0.00
2	1.88	0.00	0.00	0.00	0.00	0.00
3	2.54	1.40	0.00	0.00	0.00	0.00
4	3.05	0.00	88.91	0.00	0.00	0.00
6	1.44	0.00	0.59	0.00	0.00	0.00
7	2.60	1.39	0.00	0.00	0.00	0.55
9	1.75	0.00	0.00	0.45	0.00	0.56
10	2.95	0.00	79.12	0.00	0.00	0.00
32	4.36	0.00	0.00	0.00	0.00	0.00
33	2.17	0.00	1.50	0.00	0.00	0.00
34	6.48	0.00	0.00	0.00	0.60	0.00
35	2.01	0.00	3.94	0.00	0.00	0.00
37	5.29	4.51	3.37	0.56	0.00	0.00
38	2.21	0.00	3.51	0.00	0.39	0.00
39	4.20	0.00	0.00	0.00	0.00	0.89
40	6.72	0.00	0.00	0.00	0.00	0.00
41	5.39	3.89	3.23	0.00	0.00	0.00
42	2.17	0.00	1.54	0.00	0.00	0.00

Abbreviations: ISU, ISAC standard units for IgE; CCDs, carbohydrates cross-reactive determinants; MUXF3, the CCD-marker included in ImmunoCAP ISAC; nCup a 1, native Cupressaceae allergen component from *Cupressus arizonica*, containing CCD-type glycosylation; Mus m 1, Mus m 1 allergen; Equ c 1, lipocalin allergen from *Equus caballus*; Fel d 4, lipocalin allergen from *Felix domesticus*; Can f 1, lipocalin allergen from *Canis familiaris.*

**Table 2:** Positive sera reactivity to recombinant and urinary Mus m 1 samples, assessed by ELISA.

Antigen	* Reactive Sera (%)	^§^ Non-Reactive Sera (No.)
rMus m 1.0102	88.9	1, 6
^a^ nMus m 1_lab	88.9	1, 2
^b^ nMus m 1_comm.	76.4	2, 6, 9, 33

^a^ Urinary nMus m 1 laboratory preparation, ^b^ Urinary nMus m 1 commercial preparation. * O.D._450nm_ was higher than the mean absorbance of control sera; ^§^ O.D._450nm_ was lower than the mean absorbance of control sera.

**Table 3:** Test accuracy parameters for indirect ELISA.

	AUC	*p*-Value *	Threshold **	Sensitivity	Specificity
rMus m 1.0102	0.87	2.22E-4	0.075	0.89	0.75
Mus m 1_lab	0.89	7.90E-4	0.149	0.78	0.90
Mus m 1_comm.	0.77	0.021	0.082	0.76	0.80

* Asymptotic *p*-value under the null hypothesis that AUC = 0.5 vs. alternative hypothesis. ** Threshold value of O.D._450nm_ for mouse allergy diagnosis by indirect ELISA.

**Table 4:** Intra-assay and inter-assay coefficients of variation of ELISA based on rMus m 1.0102.

Serum No. (Specific IgE Level, ISU)	Intra-Assay CV			Inter-Assay CV		
	Mean O.D.	SD	CV%	Mean O.D.	SD	CV%
34 (6.48)	0.70	0.01	1.8	0.73	0.03	4.1
39 (4.20)	4.89	0.32	6.5	4.84	0.39	8.0
2 (1.88)	0.21	0.01	3.5	0.22	0.02	9.1

## Data Availability

Not applicable.

## Supplementary Materials

The following supporting information can be downloaded at: https://www.mdpi.com/article/10.3390/ijms24021193/s1.
