# Supplementary material for: Component-Resolved Diagnosis Based on a Recombinant Variant of Mus m 1 Lipocalin Allergen
Source: Int J Mol Sci. 2023 Jan 7;24(2):1193. doi: 10.3390/ijms24021193 (PMC9862564; doi:10.3390/ijms24021193)
Supplement: Supplementary file 1 [file ijms-24-01193-s001.zip › ijms-2084523-supplementary.pdf]

SUPPLEMENTARY MATERIAL TO:

Research Article

Component-resolved diagnosis based on a recombinant variant of Mus m 1 lipocalin allergen

Elena Ferrari [1]*, Daniela Breda [2], Alberto Spisni [1] and Samuele E. Burastero [2]

[1] Department of Medicine and Surgery, University of Parma, Parma, Italy
[2] Division of Immunology, Transplantation and Infectious Diseases, San Raffaele Scientific Institute, Milan, Italy
* Correspondence: elena.ferrari@unipr.it

CONTENTS (order of citation):

Figure S1. Amino acid sequence alignment of *central* mouse Major Urinary Proteins.

Figure S2. Amino acid sequence alignment of Mus m 1.0102 allergen (a *central* Major Urinary Protein) and *peripheral* Major Urinary Proteins.

Figure S3. Graphical output of the B-cell epitope prediction by Discotope 2.0.

Table S1. List of the residues predicted by DiscoTope 2.0 as parts of B-cell epitopes

Table S2. List of residue scores obtained by SEPPA 3.0

Figure S4. Conformational epitopes predicted by SEPPA 3.0.

Figure S5. Protein characterization.

Table S3. Correlation analysis of indirect ELISA data

Figure S6. ROC curves of ELISA data based on rMus m 1.0102 and urinary nMus m 1 allergens (laboratory and commercial preparations).

```
CLUSTAL O(1.2.4) multiple sequence alignment


EMBOSS_001                 -------------------EEASSTGRNFNVEKINGEWHTIILASDKREKIEDNGNFRLF      41
sp|P11589|MUP2_MOUSE       -MKMLLLLCLGLTLVCVHAEEASSTGRNFNVEKINGEWHTIILASDKREKIEDNGNFRLF      59
sp|P04938|MUP11_MOUSE      MKMLLLLLCLGLTLVCVHAEEASSTGRNFNVEKINGEWHTIILASDKREKIEDNGNFRLF      60
sp|P11588|MUP1_MOUSE       -MKMLLLLCLGLTLVCVHAEEASSTGRNFNVEKINGEWHTIILASDKREKIEDNGNFRLF      59
tr|A9R9W0|A9R9W0_MOUSE     -MKMLLLLCLGLTLVCVHAEEASSTGRNFNVQKINGEWHTIILASDKREKIEDNGNFRLF      59
tr|A2CEK7|A2CEK7_MOUSE     -MKMLLLLCLGLTLVCVHAEEASSTGRNFNVEKINGEWHTIILASDKREKIEDNGNFRLF      59
tr|Q58EV3|Q58EV3_MOUSE     -MKMLLLLCLGLTLVCVHAEEASSTGRNFNVEKINGEWHTIILASDKREKIEDNGNFRLF      59
tr|A2AKN8|A2AKN8_MOUSE     -MKMLLLLCVGLTLVCVHAEEASSTGRNFNVEKINGEWHTIILASDKREKIEDNGNFRLF      59
sp|B5X0G2|MUP17_MOUSE      -MKMLLLLCLGLTLVCVHAEEASSTGRNFNVEKINGEWHTIILASDKREKIEEHGNFRLF      59
tr|A2CEK6|A2CEK6_MOUSE     -MKMLLLLCLGLTLVCVHAEEASSTGRNFNVEKINGEWHTIILASDKREKIEEHGNFRLF      59
                                              ************:********************::******

EMBOSS_001                 LEQIHVLEKSLVLKFHTVRDEECSELSMVADKTEKAGEYSVTYDGFNTFTIPKTDYDNFL      101
sp|P11589|MUP2_MOUSE       LEQIHVLEKSLVLKFHTVRDEECSELSMVADKTEKAGEYSVTYDGFNTFTIPKTDYDNFL      119
sp|P04938|MUP11_MOUSE      LEQIHVLENSLVLKFHTVRDEECSELSMVADKTEKAGEYSVTYDGFNTFTIPKTDYDNFL      120
sp|P11588|MUP1_MOUSE       LEQIHVLENSLVLKFHTVRDEECSELSMVADKTEKAGEYSVTYDGFNTFTIPKTDYDNFL      119
tr|A9R9W0|A9R9W0_MOUSE     LEQIHVLENSLVLKFHTVRDEECSELSMVADKTEKAGEYSVTYDGFNTFTIPKTDYDNFL      119
tr|A2CEK7|A2CEK7_MOUSE     LEQIRVLENSLVLKFHTVRDEECSELSMVADKTEKAGEYSVTYDGFNTFTIPKTDYDNFL      119
tr|Q58EV3|Q58EV3_MOUSE     LEQIHVLENSLVLKVHTVRDEECSELSMVADKTEKAGEYSVTYDGFNTFTIPKTDYDNFL      119
tr|A2AKN8|A2AKN8_MOUSE     LEQIRVLENSLVLKVHTVRDEECSELSMVADKTEKAGEYSVTYDGFNTFTIPKTDYDNFL      119
sp|B5X0G2|MUP17_MOUSE      LEQIHVLENSLVLKVHTVRDEECSELSMVADKTEKAGEYSVTYDGFNTFTIPKTDYDNFL      119
tr|A2CEK6|A2CEK6_MOUSE     LEQIHVLENSLVLKVHTVRDEECSELSMVADKTEKAGKYSVTYDGFNTFTIPKTDYDNFL      119
                           ****:***:*****.***********************:*********************

EMBOSS_001                 MAHLINEKDGETFQLMGLYGREPDLSSDIKERFAQLCEEHGILRENIIDLSNANRCLQAR      161
sp|P11589|MUP2_MOUSE       MAHLINEKDGETFQLMGLYGREPDLSSDIKERFAKLCEEHGILRENIIDLSNANRCLQAR      179
sp|P04938|MUP11_MOUSE      MAHLINEKDGETFQLMGLYGREPDLSSDIKERFAQLCEEHGILRENIIDLSNANRCLQAR      180
sp|P11588|MUP1_MOUSE       MAHLINEKDGETFQLMGLYGREPDLSSDIKERFAQLCEKHGILRENIIDLSNANRCLQAR      179
tr|A9R9W0|A9R9W0_MOUSE     MAHLINEKDGETFQLMGLYGREPDLSSDIKERFAQLCEKHGILRENIIDLSNANRCLQAR      179
tr|A2CEK7|A2CEK7_MOUSE     MAHLINEKDGETFQLMGLYGREPDLSSDIKERFAQLCEEHGILRENIIDLSNANRCLQAR      179
tr|Q58EV3|Q58EV3_MOUSE     MAHLINEKDGETFQLMGLYGREPDLSSDIKERFAQLCEKHGILRENIIDLSNANRCLQAR      179
tr|A2AKN8|A2AKN8_MOUSE     MAHLINEKDGETFQLMGLYGREPDLSSDIKERFAQLCEEHGILRENIIDLSNANRCLQAR      179
sp|B5X0G2|MUP17_MOUSE      MAHLINEKDGETFQLMGLYGREPDLSSDIKERFAQLCEEHGILRENIIDLSNANRCLQAR      179
tr|A2CEK6|A2CEK6_MOUSE     MAHLINEKDGETFQLMGLYGREPDLSSDIKERFAQLCEEHGILRENIIDLSNANRCLQAR      179
                           **********************************:***:*********************

EMBOSS_001                 E*   162
sp|P11589|MUP2_MOUSE       E-   180
sp|P04938|MUP11_MOUSE      E-   181
sp|P11588|MUP1_MOUSE       E-   180
tr|A9R9W0|A9R9W0_MOUSE     E-   180
tr|A2CEK7|A2CEK7_MOUSE     E-   180
tr|Q58EV3|Q58EV3_MOUSE     E-   180
tr|A2AKN8|A2AKN8_MOUSE     E-   180
sp|B5X0G2|MUP17_MOUSE      E-   180
tr|A2CEK6|A2CEK6_MOUSE     E-   180
                           *
```

Figure S1. Amino acid sequence alignment of *central* mouse Major Urinary Proteins. Align tool, running the Clustal Omega algorithm to find sections of identity/similarity, was used for multiple sequence alignment. Consensus symbols: asterisk indicates a position which has a fully conserved residue; colon indicates a position which has a substitution between amino acids with similar properties (roughly equivalent to scoring > 0.5 in the Gonnet PAM 250 matrix [27]); period indicates a position which has almost one substitution between amino acids of weakly similar properties (roughly equivalent to scoring ≤ 0.5 and >0 in the Gonnet PAM 250 matrix). Each protein is represented by its accession number in UniProtKB database, while the query protein Mus m 1.0102 is indicated as EMBOSS_001 (dashed line at the beginning of the sequence indicates omission of the secretion signal).

```
CLUSTAL O(1.2.4) multiple sequence alignment


EMBOSS_001                      ----------------------EEASSTGRNFNVEKINGEWHTIILASDKREKIEDNGNF      38
sp|Q5FW60|MUP20_MOUSE           ---MKLLVLLLCLGLTLVCVHAEEASSMERNFNVEKINGEWYTIMLATDKREKIEEHGSM      57
tr|A2AV72|A2AV72_MOUSE          -----MKLLLLCLGLILVCVHAEEASSMGRNFNVEKINGEWYTIILASDKRAKIEEHGIM      55
sp|P11591|MUP5_MOUSE            ----MKLLLLLLCLELTLVYVHAEEASSEGQNLNVEKINGKWFSILLASDKREKIEEHGTM      56
sp|P11590|MUP4_MOUSE            ------MKLLLCLGLTLVCIHAEEATSKGQNLNVEKINGEWFSILLASDKREKIEEHGSM      54
tr|Q3KQQ2|Q3KQQ2_MOUSE          MKLLLPLLLLLCLELTLVCIHAEESSSMERNFNVEQISGYWFSIAEASDEREKIEEHGSM      60
sp|P04939|MUP3_MOUSE            MKLLLPLLLLLCLELTLVCIHAEESSSMERNFNVEQISGYWFSIAEASYEREKIEEHGSM      60
tr|Q80YX8|Q80YX8_MOUSE          ---MKLLLLLLCLGLTIVCIQAEEYSSMGRNFNVEQISGYWFSIAEASDEREKIEEHGSM      57
sp|P02762|MUP6_MOUSE            ----MKMLLLLCLGLTLVCVHAEEASSTGRNFNVEKINGEWHTIILASDKREKIEDNGNF      56
                                                      ** :*  :*:***:*.* *.:*  *: :* ***::* :

EMBOSS_001                      RLFLEQIHVLEKSLVLKFHTVRDEECSELSMVADKTEKAGEYSVTYDGFNTFTIPKTDYD      98
sp|Q5FW60|MUP20_MOUSE           RVFVEYIHVLENSLALKFHIIINEECSEIFLVADKTEKAGEYSVTYDGSNTFTILKTDYD      117
tr|A2AV72|A2AV72_MOUSE          RLFVEHIHVLENSLGFKFHTVIDEECSEIFLVADKTEKAGEYSVTYDGFKKFTVLKTDYD      115
sp|P11591|MUP5_MOUSE            RVFVEHIDVLENSLAFKFHTVIDEECTEIYLVADKTEKAGEYSVTYDGFNTFTILKTDYD      116
sp|P11590|MUP4_MOUSE            RVFVEHIHVLENSLAFKFHTVIDGECSEIFLVADKTEKAGEYSVMYDGFNTFTILKTDYD      114
tr|Q3KQQ2|Q3KQQ2_MOUSE          RAFVENITVLENSLVFKFHLIVNEECTEMTAIGEQTEKAGIYYMNYDGFNTFSILKTDYD      120
sp|P04939|MUP3_MOUSE            RAFVENITVLENSLVFKFHLIVNEECTEMTAIGEQTEKAGIYYMNYDGFNTFSILKTDYD      120
tr|Q80YX8|Q80YX8_MOUSE          RAFVENITVLENSLVFKFHFIVNEECTEMTLIGEETEKAGIYYLNYDGFNTFTILKTDYD      117
sp|P02762|MUP6_MOUSE            RLFLEQIHVLENSLVLKFHTVRDEECSELSMVADKTEKAGEYSVTYDGFNTFTIPKTDYD      116
                                * *:* * ***:** :*** : : **:*:  :.::***** * : *** :.*:: *****

EMBOSS_001                      NFLMAHLINEKDGETFQLMGLYGREPDLSSDIKERFAQLCEEHGILRENIIDLSNANRCL      158
sp|Q5FW60|MUP20_MOUSE           NYIMIHLINKKDGETFQLMELYGREPDLSSDIKEKFAQLSEEHGIVRENIIDLTNANRCL      177
tr|A2AV72|A2AV72_MOUSE          NYIMFHLINEMNGETFQLMSLYGREPDLNSDIKEKFVKLCEEHGIIRENIIDFTKTNRCL      175
sp|P11591|MUP5_MOUSE            NYIMFHLINKKDEENFQLMELFGREPDLSSDIKEKFAKLCEEHGIVRENIIDLSNANRCL      176
sp|P11590|MUP4_MOUSE            NYIMFHLINEKDGKTFQLMELYGRKADLNSDIKEKFVKLCEEHGIIKENIIDLTKTNRCL      174
tr|Q3KQQ2|Q3KQQ2_MOUSE          NYIMIHLINKKDGKTFQLMELYGREPDLSLDIKEKFAKLCEEHGIIRENIIDLTNVNRCL      180
sp|P04939|MUP3_MOUSE            NYIMIHLINKKDGKTFQLMELYGREPDLSLDIKEKFAKLCEEHGIIRENIIDLTNVNRCL      180
tr|Q80YX8|Q80YX8_MOUSE          NYIMIYLINEKDGETFQLMELYGREPYLSLDIKEKFAKLCEEHGIIRENIIDLTNVNRCL      177
sp|P02762|MUP6_MOUSE            NFLMAHLINEKDGETFQLMGLYGREPDLSSDIKERFAQLCEEHGILRENIIDLSNANRCL      176
                                *::* :***: : :.**** *:**:  *. ****:*.:*.*****::*****:::.****

EMBOSS_001                      QARE*     162
sp|Q5FW60|MUP20_MOUSE           EARE-     181
tr|A2AV72|A2AV72_MOUSE          QARE-     179
sp|P11591|MUP5_MOUSE            QARE-     180
sp|P11590|MUP4_MOUSE            KARE-     178
tr|Q3KQQ2|Q3KQQ2_MOUSE          EARE-     184
sp|P04939|MUP3_MOUSE            EARE-     184
tr|Q80YX8|Q80YX8_MOUSE          EARE-     181
sp|P02762|MUP6_MOUSE            QARE-     180
                                :***
```

Figure S2. Amino acid sequence alignment of Mus m 1.0102 allergen (a *central* Major Urinary Protein) and *peripheral* Major Urinary Proteins. Align tool, running the Clustal Omega algorithm to find sections of identity/similarity, was used for multiple sequence alignment. Consensus symbols: asterisk indicates a position which has a single, fully conserved residue; colon indicates a position which has at least one substitution between amino acids with similar properties (roughly equivalent to scoring > 0.5 in the Gonnet PAM 250 matrix [27]); period indicates a position which has almost one substitution between amino acids of weakly similar properties (roughly equivalent to scoring ≤ 0.5 and >0 in the Gonnet PAM 250 matrix); absence of a symbol corresponds to non-conservative substitutions. Each protein is represented by its accession number in UniProtKB database, while the query protein rMus m 1.0102 is indicated as EMBOSS_001 (dashed line at the beginning of the sequence indicates omission of the secretion signal).

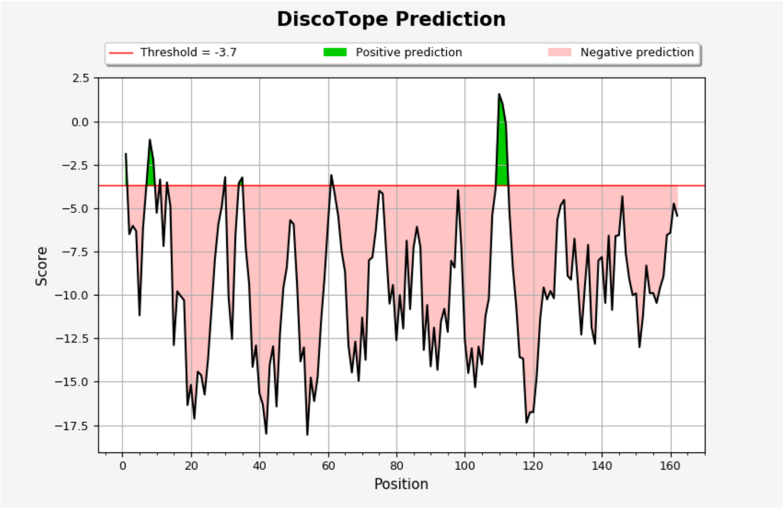

Figure S3. Graphical output of the B-cell epitope prediction by Discotope 2.0.

Table S1. List of the residues predicted by Discotope 2.0 as parts of B-cell epitopes

Residue	Contact No.	Propensity score	Discotope score
E1*	8	-1.089	-1.884
G7	4	-3.642	-3.684
R8	8	-0.156	-1.058
N9	8	-1.465	-2.217
N11	10	-2.486	-3.351
E13	1	-3.848	-3.521
E30	4	-3.115	-3.217
D34	1	-3.884	-3.553
N35	3	-3.262	-3.232
D61	0	-3.502	-3.1
D110	1	1.903	1.569
G111	0	1.136	1.006
E112	4	0.293	-0.201

* The color code corresponds to epitope highlighting in Figure 1C and 1D.

Table S2. List of residue scores obtained by SEPPA 3.0 server

chainID	resSeq	resName	score*	location
A	1	GLU	0.065	surface
A	2	GLU	0.056	surface
A	3	ALA	0.056	surface
A	4	SER	0.069	surface
A	5	SER	0.057	surface
A	6	THR	0.064	surface
A	7	GLY	0.078	surface
**A**	**8**	**ARG**	**0.094**	**surface** $
A	9	ASN	0.069	surface
A	10	PHE	0.070	surface
A	11	ASN	0.072	surface
A	12	VAL	0.055	surface
A	13	GLU	0.052	surface
A	14	LYS	0.062	surface
A	15	ILE	0.000	core
A	16	ASN	0.048	surface
A	17	GLY	0.046	surface
A	18	GLU	0.057	surface
A	19	TRP	0.000	core
A	20	HIS	0.071	surface
A	21	THR	0.000	core
A	22	ILE	0.000	core
A	23	ILE	0.051	surface
A	24	LEU	0.041	surface
A	25	ALA	0.000	core
A	26	SER	0.000	core
A	27	ASP	0.049	surface
A	28	LYS	0.071	surface
A	29	ARG	0.059	surface
A	30	GLU	0.070	surface
A	31	LYS	0.067	surface
A	32	ILE	0.000	core
A	33	GLU	0.068	surface
A	34	ASP	0.077	surface
A	35	ASN	0.079	surface
A	36	GLY	0.062	surface
A	37	ASN	0.054	surface
A	38	PHE	0.048	surface
A	39	ARG	0.066	surface
A	40	LEU	0.042	surface
A	41	PHE	0.061	surface
A	42	LEU	0.039	surface
A	43	GLU	0.041	surface
A	44	GLN	0.037	surface
A	45	ILE	0.038	surface
A	46	HIS	0.041	surface
A	47	VAL	0.048	surface
A	48	LEU	0.056	surface
A	49	GLU	0.060	surface
A	50	LYS	0.059	surface
A	51	SER	0.057	surface
A	52	LEU	0.000	core
A	53	VAL	0.040	surface
A	54	LEU	0.033	surface
A	55	LYS	0.038	surface
A	56	PHE	0.040	surface
A	57	HIS	0.064	surface
A	58	THR	0.086	surface

**A**	**59**	**VAL**	**0.129**	**surface**	
**A**	**60**	**ARG**	**0.141**	**surface**	
**A**	**61**	**ASP**	**0.197**	**surface**	$
**A**	**62**	**GLU**	**0.171**	**surface**	
**A**	**63**	**GLU**	**0.153**	**surface**	
**A**	**64**	**CYS**	**0.130**	**surface**	
**A**	**65**	**SER**	**0.099**	**surface**	
A	66	GLU	0.055	surface	
A	67	LEU	0.043	surface	
A	68	SER	0.035	surface	
A	69	MET	0.035	surface	
A	70	VAL	0.046	surface	
A	71	ALA	0.000	core	
A	72	ASP	0.055	surface	
A	73	LYS	0.061	surface	
A	74	THR	0.057	surface	
A	75	GLU	0.060	surface	
A	76	LYS	0.068	surface	
A	77	ALA	0.064	surface	
A	78	GLY	0.000	core	
A	79	GLU	0.053	surface	
A	80	TYR	0.000	core	
A	81	SER	0.049	surface	
A	82	VAL	0.044	surface	
A	83	THR	0.050	surface	
A	84	TYR	0.047	surface	
A	85	ASP	0.069	surface	
**A**	**86**	**GLY**	**0.120**	**surface**	
A	87	PHE	0.065	surface	
A	88	ASN	0.000	core	
A	89	THR	0.046	surface	
A	90	PHE	0.034	surface	
A	91	THR	0.036	surface	
A	92	ILE	0.038	surface	
A	93	PRO	0.040	surface	
A	94	LYS	0.039	surface	
A	95	THR	0.000	core	
A	96	ASP	0.043	surface	
A	97	TYR	0.048	surface	
A	98	ASP	0.047	surface	
A	99	ASN	0.049	surface	
A	100	PHE	0.048	surface	
A	101	LEU	0.036	surface	
A	102	MET	0.042	surface	
A	103	ALA	0.031	surface	
A	104	HIS	0.033	surface	
A	105	LEU	0.034	surface	
A	106	ILE	0.053	surface	
A	107	ASN	0.000	core	
**A**	**108**	**GLU**	**0.144**	**surface**	
**A**	**109**	**LYS**	**0.142**	**surface**	
**A**	**110**	**ASP**	**0.169**	**surface**	$
**A**	**111**	**GLY**	**0.220**	**surface**	$
**A**	**112**	**GLU**	**0.196**	**surface**	$
**A**	**113**	**THR**	**0.128**	**surface**	
A	114	PHE	0.081	surface	
A	115	GLN	0.052	surface	
A	116	LEU	0.039	surface	
A	117	MET	0.000	core	
A	118	GLY	0.000	core	
A	119	LEU	0.000	core	
A	120	TYR	0.039	surface	
A	121	GLY	0.000	core	

A	122	ARG	0.054	surface
A	123	GLU	0.073	surface
**A**	**124**	**PRO**	**0.093**	**surface**
**A**	**125**	**ASP**	**0.104**	**surface**
A	126	LEU	0.069	surface
A	127	SER	0.080	surface
A	128	SER	0.075	surface
A	129	ASP	0.075	surface
A	130	ILE	0.063	surface
A	131	LYS	0.072	surface
A	132	GLU	0.067	surface
A	133	ARG	0.060	surface
A	134	PHE	0.000	core
A	135	ALA	0.058	surface
A	136	GLN	0.056	surface
A	137	LEU	0.047	surface
A	138	CYS	0.000	core
A	139	GLU	0.046	surface
A	140	GLU	0.046	surface
A	141	HIS	0.043	surface
A	142	GLY	0.048	surface
A	143	ILE	0.046	surface
A	144	LEU	0.050	surface
A	145	ARG	0.048	surface
A	146	GLU	0.050	surface
A	147	ASN	0.050	surface
A	148	ILE	0.046	surface
A	149	ILE	0.056	surface
A	150	ASP	0.069	surface
A	151	LEU	0.065	surface
**A**	**152**	**SER**	**0.093**	**surface**
**A**	**153**	**ASN**	**0.107**	**surface**
A	154	ALA	0.074	surface
A	155	ASN	0.088	surface
**A**	**156**	**ARG**	**0.108**	**surface**
**A**	**157**	**CYS**	**0.112**	**surface**
**A**	**158**	**LEU**	**0.114**	**surface**
**A**	**159**	**GLN**	**0.138**	**surface**
**A**	**160**	**ALA**	**0.145**	**surface**
**A**	**161**	**ARG**	**0.136**	**surface**
**A**	**162**	**GLU**	**0.124**	**surface**

*Residues with a score higher than the threshold (0.089) are predicted as epitope residues (*in bold*).

§ Residues in blue, red, and green were predicted also by Discotope 2.0 as parts of B-cell epitopes (see Figure 1, epitopes I, II, III).

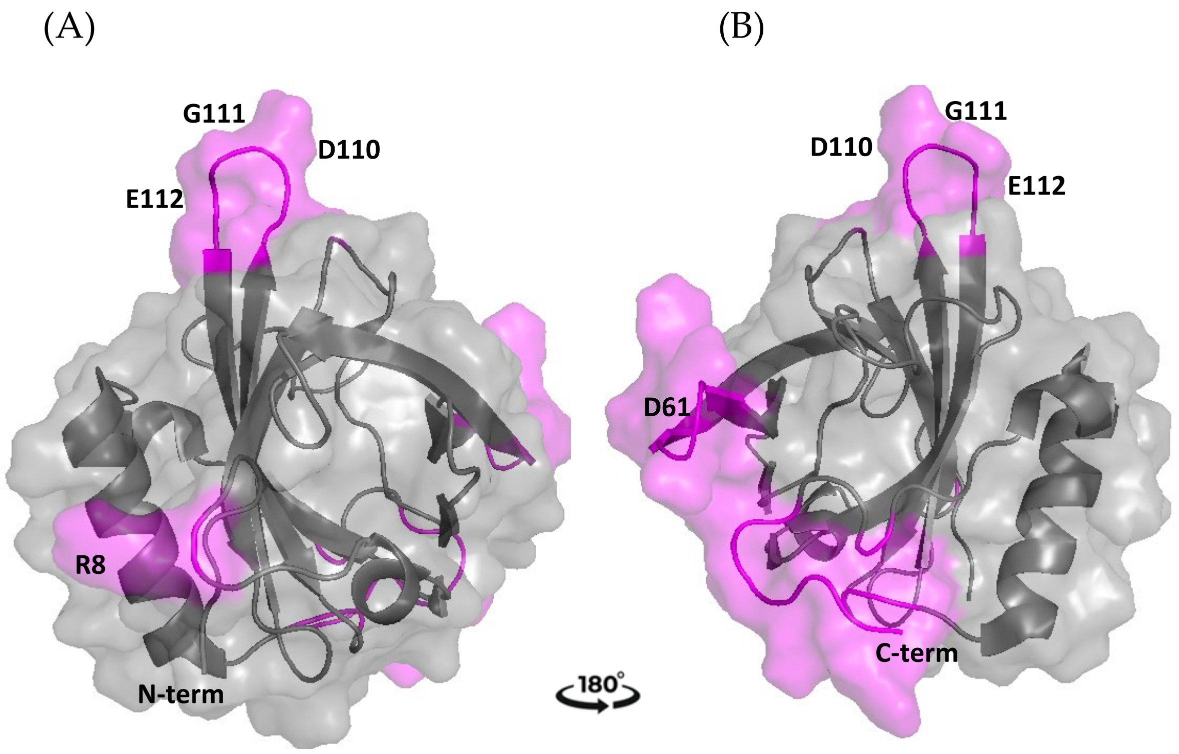

Figure S4. Conformational epitopes predicted by SEPPA 3.0. (**A**,**B**) The magenta regions of surface and cartoon representations of rMus m 1.0102 solution structure (PDB code 1df3, model 1) correspond to predicted B-cell epitopes. Labelled epitope residues (R8, D61, D110, G111, E112) were predicted also by Discotope 2.0 as parts of B-cell epitopes (Figure 1C,D). (**A**) bottom view of the central cavity, (**B**) top view of the cavity, generated by a 180° turn.

(A)

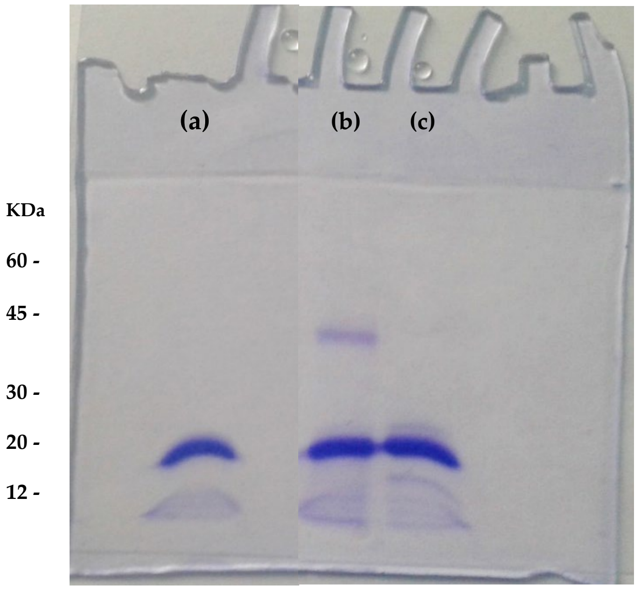

(B)

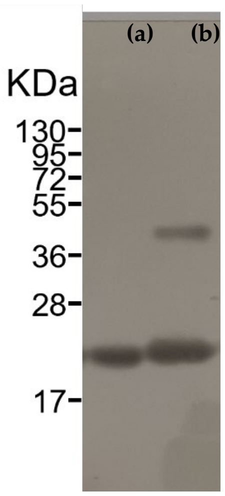

(C)

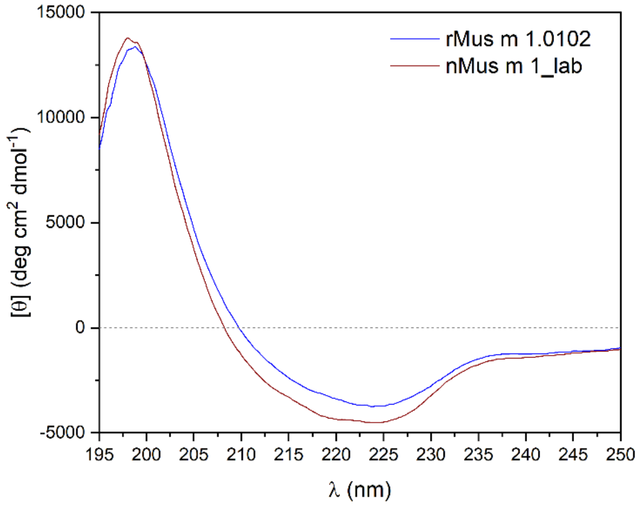

Figure S5. Protein characterization. (**A**) SDS-PAGE and (**B**) western blot analyses of rMus m 1.0102 (lanes a) , laboratory (lanes b), and commercial (Indoor Biotechnologies) (lane c) preparations of urinary nMus m 1. Apparently, the band at 40 kDa of the laboratory preparation of nMus m 1 might be ascribed to a dimer formation, possibly due to a cysteine reoxidation reaction. The low molecular weight bands appreciable at <20 kDa of nMus m 1 commercial preparation sample might be due to a partial degradation process. (**C**) CD spectra for rMus m 1.0102 and nMus m 1 laboratory preparation samples, acquired in the far-UV region.

Table S3. Correlation analysis of indirect ELISA data

Variables	Spearman corr. Coefficient	*p*-Value
- ELISA data:		
rMus m 1.0102/[a] nMus m 1	0.51496	0.02875*
rMus m 1.0102/[b] nMus m 1	0.43627	0.07999
[a] nMus m 1/[b] nMus m 1	0.64461	0.00521*
- ELISA data vs. ImmunoCAP ISAC data:		
rMus m 1.0102/Mus m 1-specific IgE	0.55005	0.01803*

* Significant *p*-value at the 0.05 level

[a] nMus m 1 laboratory preparation; [b] nMus m 1 commercial preparation.

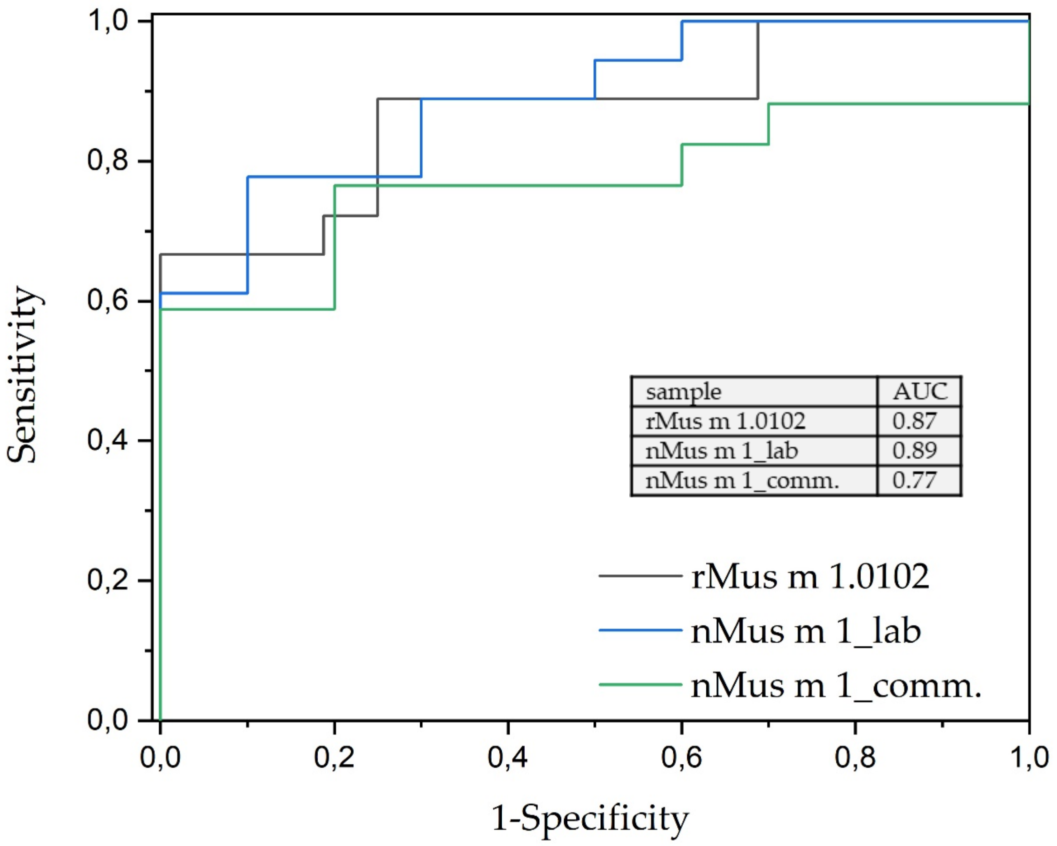

Figure S6. ROC curves of ELISA data based on rMus m 1.0102 and urinary nMus m 1 allergens (laboratory and commercial preparations). Data were obtained with control and allergic sera.
